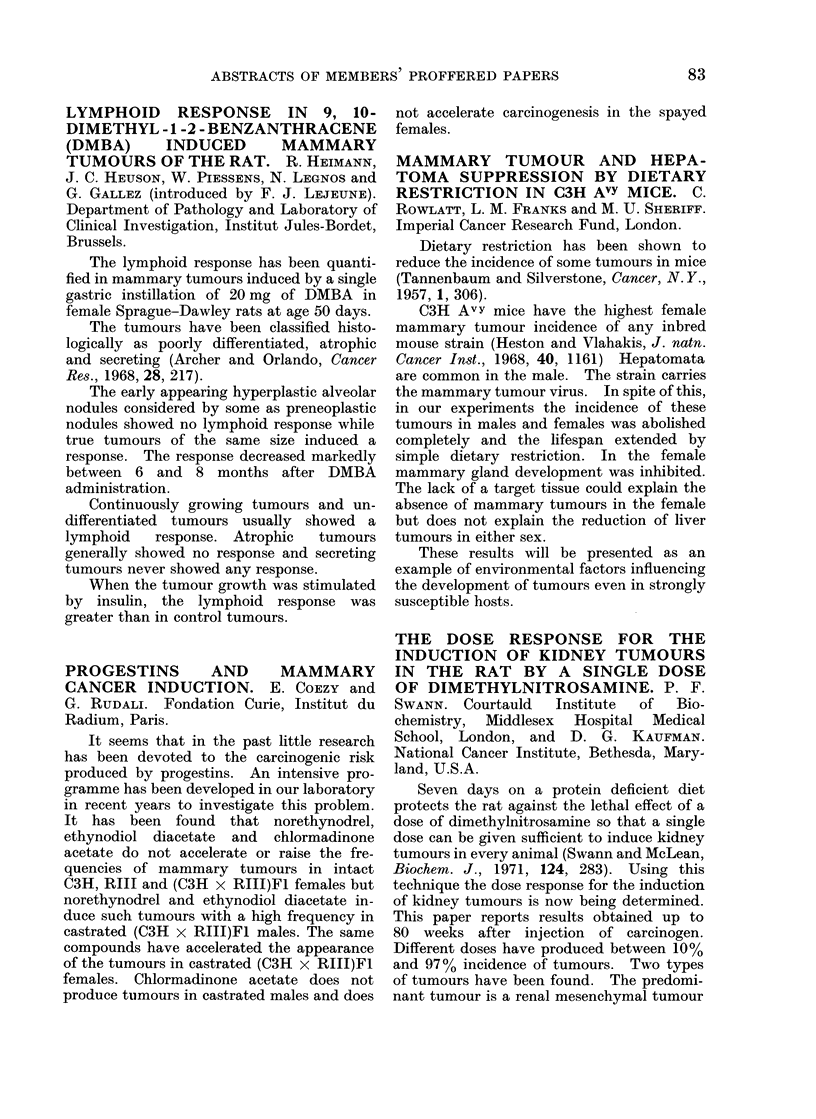# Lymphoid response in 9, 10-dimethyl-1-2-benzanthracene (DMBA) induced mammary tumours of the rat.

**DOI:** 10.1038/bjc.1973.96

**Published:** 1973-07

**Authors:** R. Heimann, J. C. Heuson, W. Piessens, N. Legnos, G. Gallez


**LYMPHOID RESPONSE IN 9, 10-DIMETHYL-1-2-BENZANTHRACENE (DMBA) INDUCED MAMMARY TUMOURS OF THE RAT.** R. HEIMANN, J. C. HEUSON, W. PIESSENS, N. LEGNOS and G. GALLEZ (introduced by F. J. LEJEUNE). Department of Pathology and Laboratory of Clinical Investigation, Institut Jules-Bordet, Brussels.

The lymphoid response has been quantified in mammary tumours induced by a single gastric instillation of 20 mg of DMBA in female Sprague–Dawley rats at age 50 days.

The tumours have been classified histologically as poorly differentiated, atrophic and secreting (Archer and Orlando, *Cancer Res.*, 1968, **28**, 217).

The early appearing hyperplastic alveolar nodules considered by some as preneoplastic nodules showed no lymphoid response while true tumours of the same size induced a response. The response decreased markedly between 6 and 8 months after DMBA administration.

Continuously growing tumours and undifferentiated tumours usually showed a lymphoid response. Atrophic tumours generally showed no response and secreting tumours never showed any response.

When the tumour growth was stimulated by insulin, the lymphoid response was greater than in control tumours.